# Pitfalls in the evaluation of *CDKN2A* copy number status in meningioma

**DOI:** 10.1007/s11060-025-05029-6

**Published:** 2025-04-14

**Authors:** Valentina Zschernack, Giuseppe Pinto, Lea L. Friker, Rebecca Klein, Tobias Goschzik, Evelyn Dörner, Andreas Waha, Christian Vokuhl, Hartmut Vatter, Torsten Pietsch

**Affiliations:** 1 Institute of Neuropathology, DGNN Brain Tumor Reference Center, University Hospital Bonn, Bonn, Germany; 2 University of Bologna, Bologna, Italy; 3 Section of Pediatric Pathology, University Hospital Bonn, Bonn, Germany; 4 Department of Neurosurgery, University Hospital Bonn, Bonn, Germany; 5 Institute of Neuropathology, University Hospital Bonn, Venusberg-Campus 1, 53127 Bonn, Germany

## Abstract

**Purpose:**

Meningiomas are the most common primary intracranial tumors, with anaplastic variants linked to a poor prognosis. *CDKN2A* deletions are key markers of malignancy and were integrated into the 2021 WHO classification for anaplastic meningiomas. Both p16 and MTAP immunohistochemistry (IHC) are employed to assess *CDKN2A* loss, though each marker has limitations in accuracy to varying degrees.

**Methods:**

This study analyzed the concordance between molecular methods - DNA methylation profiling, molecular inversion probe (MIP) analysis, targeted next-generation sequencing (NGS), and fluorescence in situ hybridization (FISH) - and protein expression of p16 and MTAP in nine anaplastic meningiomas.

**Results:**

We showed that while p16 loss correlated well, MTAP protein was still expressed in three cases despite homozygous *CDKN2A* deletions. In those three cases the *MTAP* gene was hemizygously deleted. Additionally, a FISH probe encompassing both genes generated misleading results.

**Conclusion:**

Our results suggest that MTAP IHC can be unreliable as a sole surrogate for *CDKN2A* loss in anaplastic meningioma. Quantitative copy-number analysis via high-resolution chromosomal arrays enables precise determination of *CDKN2A* deletions. Given the therapeutic implications of WHO grading, accurate molecular testing is critical. We conclude that negative p16/MTAP IHC in high-grade meningiomas should prompt molecular analysis for *CDKN2A* deletions, and MTAP IHC should not be solely relied upon for classification.

## Introduction

Meningiomas are the most common primary intracranial tumors in adults [25]. While the majority of meningiomas represent CNS WHO grade 1 tumors with a favorable prognosis, anaplastic meningioma are associated with poor survival [5, 11]. *TERT* promoter mutations and homozygous deletions of *CDKN2A*/*B* have been identified as indicators of worse prognosis and were incorporated into the 2021 WHO classification criteria for the diagnosis of anaplastic meningioma [11]. In a recent update from the cIMPACT-NOW consortium, additional molecular parameters, such as complete or segmental deletions of 1p in the presence of monosomy 22 or NF2 mutations, were introduced as markers for CNS WHO grade 2 meningioma [18]. As a result, molecular testing has become increasingly important for the classification and management of meningiomas.

In 2002, deletions in chromosome 9p21, which includes the *CDKN2A*, *CDKN2B* and *MTAP* genes, were linked to malignant progression or anaplastic phenotype in meningiomas [17]. *CDKN2A* encodes p16, a cyclin-dependent kinase inhibitor that plays a crucial role in regulating the cell cycle and is often inactivated by deletion or hypermethylation in various cancers [12]. In low-grade meningiomas, p16 is generally not expressed [21]. *MTAP* is located adjacent to *CDKN2A*/*B* on 9p21 and encodes a protein involved in the salvage synthesis of adenine and methionine from 5’-methylthioadenosine (MTA) [1].

Several studies have investigated p16 protein detection as a surrogate biomarker for homozygous *CDKN2A* deletions in brain tumors [21, 28]. Additionally, MTAP immunohistochemistry (IHC) has been reported to be a reliable surrogate for *CDKN2A* copy number status [14]. Yet, considerable variability remains in how different institutions assess and interpret *CDKN2A* status [14, 16, 19, 22]. To address this issue, we analyzed the accuracy of routinely used molecular methods - DNA methylation profiling, molecular inversion probe (MIP) analysis, targeted next-generation DNA panel sequencing (NGS), and fluorescence in situ hybridization (FISH) - in determining the genetic status of *CDKN2A* and *MTAP*, and compared the genetic data with the surrogate protein expression of p16 and MTAP in nine cases of anaplastic meningiomas.

## Methods

### Tissue samples

Tumor tissue from nine anaplastic meningiomas was analyzed at the Department of Neuropathology, University Hospital Bonn, Bonn, Germany and diagnosed according to the 2021 WHO CNS tumors classification. Immunohistochemical staining for p16 (mouse monoclonal, E6H4, Roche Diagnostics) and MTAP (mouse monoclonal, 2G4, dilution 1:25, Abnova) was performed on 4-µm thick formalin-fixed, paraffin-embedded (FFPE) tissue slide sections using an automated immunostaining system (BenchMark XT, Ventana-Roche, Mannheim, Germany). Immunohistochemical staining for p16 and MTAP was evaluated by two neuropathologists (TP and VZ).

### DNA extraction and molecular inversion probe assay

Hematoxylin-eosin (H&E) stained sections from each case were carefully reviewed prior to DNA extraction. DNA was extracted with the QIAmp DNA Mini Tissue Kit (Qiagen GmbH, Düsseldorf, Germany) following the manufacturer’s protocol. Genomic copy number alteration, including losses and gains, were evaluated using a Molecular Inversion Probe (MIP) array (OncoScan CNV Plus Array, Affymetrix, Santa Clara, CA, USA). The MIP array consists of 335,000 inversion probes (version V2.0) with a median probe spacing of 2.4 kb, including 34 probes targeting the *CDKN2A* gene locus. MIP analysis was conducted as previously described [6, 26]. Raw data were processed using the Nexus Copy Number 10.0 Discovery Edition software (Bionano Genomics, San Diego, CA, USA). For copy number and loss of heterozygosity (LOH) estimations, the SNP-FASST2 segmentation algorithm, as provided by the manufacturer, was applied.

### Methylation analysis

Tumor DNA was bisulfite-treated and subsequently analyzed using the Infinium Human Methylation EPIC2 (935k) array (Illumina, San Diego, CA, USA), following the manufacturer’s protocol. Methylation data generated from the array were processed as previously described in order to generate copy number profiles [8–10]. Tumors were classified into methylation groups using the Heidelberg Brain Tumor Classifier, version 12.8.

### Next-generation sequencing

Next-generation sequencing was conducted on a MiSeq device (Illumina, San Diego, CA, USA) following the manufacturer’s protocol for Illumina DNA Prep with enrichment library preparation. Copy number alterations were analyzed using the OncoCNV caller v1.2.0 [3] and Nexus 10.0 software. All 7 exons of *CDKN2A* were covered by the NGS panel and the mean of the seven exons was calculated by the Nexus software. For the NGS based detection of copy number alterations, a cohort of 8 normal tissue samples from FFPE material was analyzed by the same technology and a baseline was calculated from the .bam files of the 8 samples. The thresholds for the copy number alterations were defined as follows: maintenance of heterozygosity >-0,7; LOH <-0,7; homozygous deletion < -1,5.

### Fluorescence in situ hybridization (FISH) analysis

Multicolor interphase FISH analysis was conducted following the manufacturer’s protocols. One probe targeted the *CDKN2A* locus specifically (XL CDKN2A Locus -Specific Probe, Meta Systems, Altlussheim, Germany), while in a second FISH analysis the probe simultaneously targeted both *MTAP* and *CDKN2A/B* (Zytolight, SPEC CDKN2A/CEN 9 Dual Color Probe, Zytovision, Bremerhaven, Germany).

## Results

On histological examination, all tumors demonstrated signs of atypia such as necrosis (7/9), pattern loss (9/9), and prominent nucleoli (9/9). Increased proliferative activity (Ki67 index > 15%) and elevated mitotic count (range 7.8–22.1 mitoses/mm^2^) were found in all nine tumors. One tumor (case #8) exhibited a *TERT* promoter mutation (C228T), which was detected through NGS analysis. None of the tumors corresponded to a CNS WHO grade 1 meningioma based on histological findings (detailed information in Table 1).

**Table 1:**
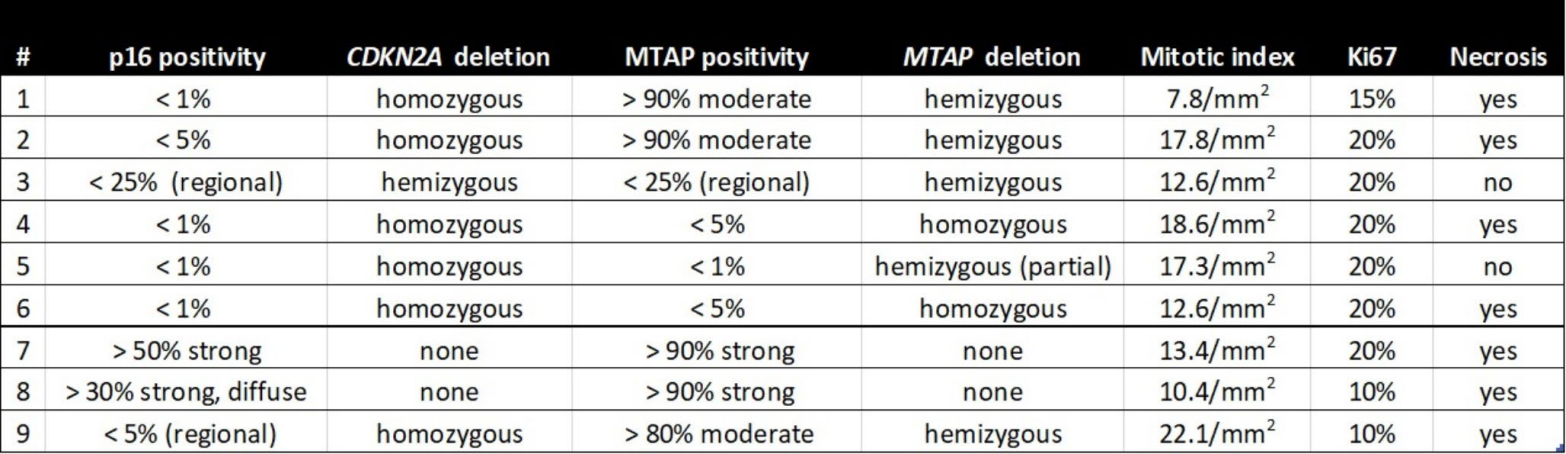
Summary of p16 and MTAP immunohistochemical analysis results, histological characteristics, and proliferation and mitotic indices for all cases

**Fig. 1:**
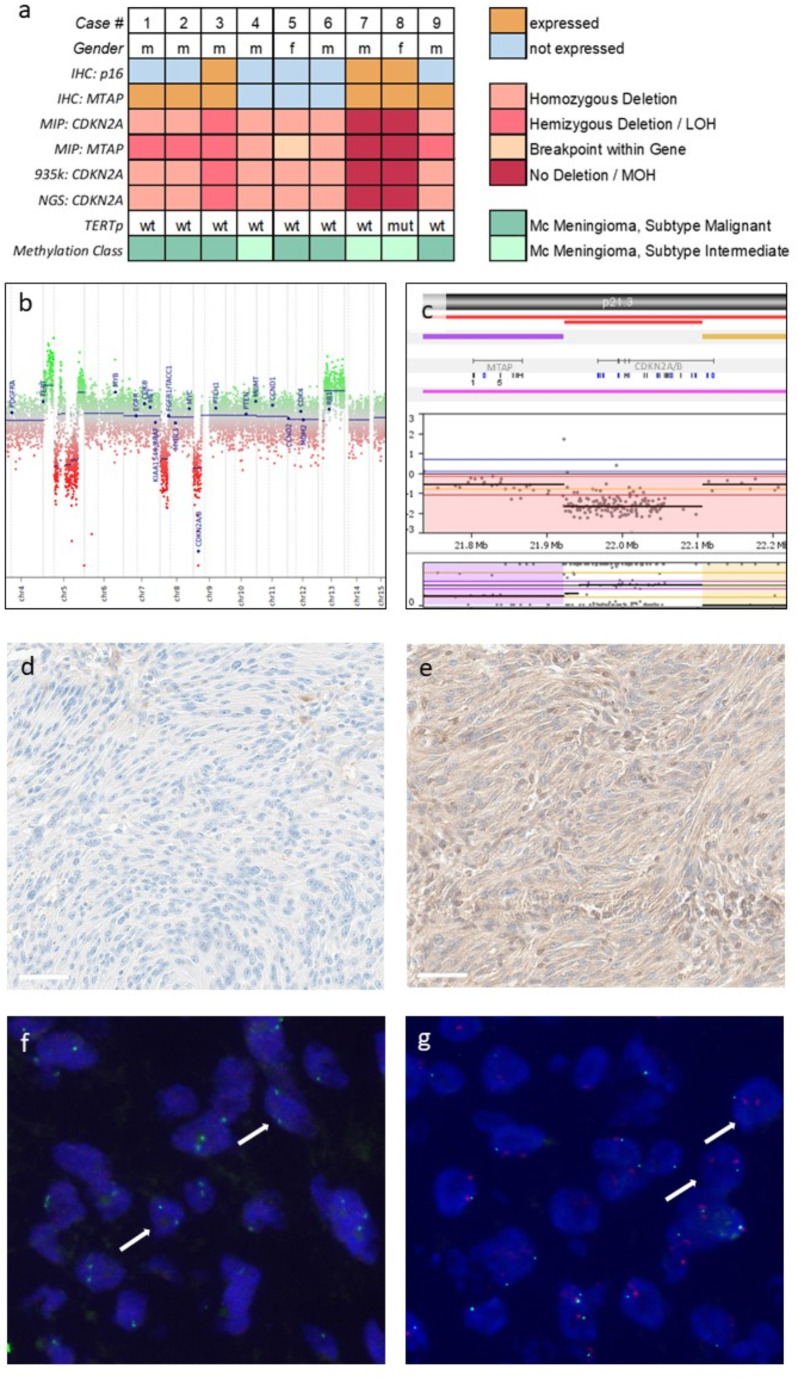
**a** Summary of the results of immunohistochemical and molecular analyses. LOH loss of heterozygosity, MOH maintenance of heterozygosity, Mc methylation class, wt wildtype, mut mutant. **b** Copy number plot of case #1 inferred from the methylation data showing a focal deletion in the *CDKN2A* region. **c***MTAP* and *CDKN2A/B* copy-number status (upper part) and allele ratio (lower part) from molecular inversion probe (MIP) array of case #1 with a breakpoint between the genes. **d** Immunohistochemistry for p16 and **e** MTAP of case #1, showing no expression of p16 and positivity for MTAP. **f** Image of a FISH analysis with a probe targeting *CDKN2A* only (two green centromere signals but no red *CDKN2A* target signal due to the homozygous deletion, arrows). **g** Image of a FISH analysis with a probe covering both *MTAP* and *CDKN2A* showing two red centromere signals and one green target signal in most of the nuclei indicating that the probe is binding to the single remaining *MTAP* allele (arrows) (63x magnification). *scale bars* in d and e: 60 μm

Results of copy number status deduced from MIP, NGS and methylation analyses regarding the estimated *CDKN2A* status were consistent. In six cases, a focal homozygous loss of the region assigned as *CDKN2A* was detected. However, MIP revealed that three of these cases (#1, #2, and #9) exhibited a breakpoint between the *MTAP* and *CDKN2A* genes, resulting in a hemizygous deletion of the *MTAP* gene only (example of #1 in Fig. 1b, c). One case with a breakpoint within the *MTAP* gene (just after exon 1; case #5, not shown) was also identified. The remaining two of these six cases with homozygous *CDKN2A* deletion showed larger homozygous deletions spanning the complete *MTAP* and *CDKN2A/B* region. Three out of nine cases showed either a hemizygous deletion of both *MTAP* and *CDKN2A* (#3) or no deletion of chromosome 9p21 at all (#7, #8).

In cases with homozygous *CDKN2A* and *MTAP* deletion, tumors exhibited cytoplasmic/nuclear positivity for p16 and MTAP in fewer than 5% of overall cells. Of note, the three cases with a breakpoint between *MTAP* and *CDKN2A* showed discordant IHC results, with positive MTAP protein expression and negative p16 staining (examples of index case #1 in Fig. 1d, e), which can be explained by their respective copy number status. Case #5 showed neither MTAP nor p16 protein expression (as in this case *MTAP* was also almost completely deleted). In those two cases without *CDKN2A* and *MTAP* deletion (#7, #8), protein expression of both p16 and MTAP was retained. Case #3, which displayed hemizygous loss of *CDKN2A/MTAP* on MIP, demonstrated focal positivity for both p16 and MTAP in less than 25% of the tumor tissue.

FISH was performed on the indicator case #1 using two different probes: one targeting a region on 9p21.3 that specifically binds to *CDKN2A* (XL CDKN2A Locus -Specific Probe), and another commercially available probe that spans a broader region, including both *CDKN2A* and *MTAP* (Zytolight). The *CDKN2A* specific probe targeting the smaller region on 9p21.3 showed no signal for *CDKN2A* (no red signal; Fig. 1f) as expected. In contrast, FISH analysis using the commercial probe that binds to a larger region on 9p21.3 covering *MTAP* and *CDKN2A* detected both centromeric signals (red) and a target signal (green; Fig. 1g).

**Fig. 2:**
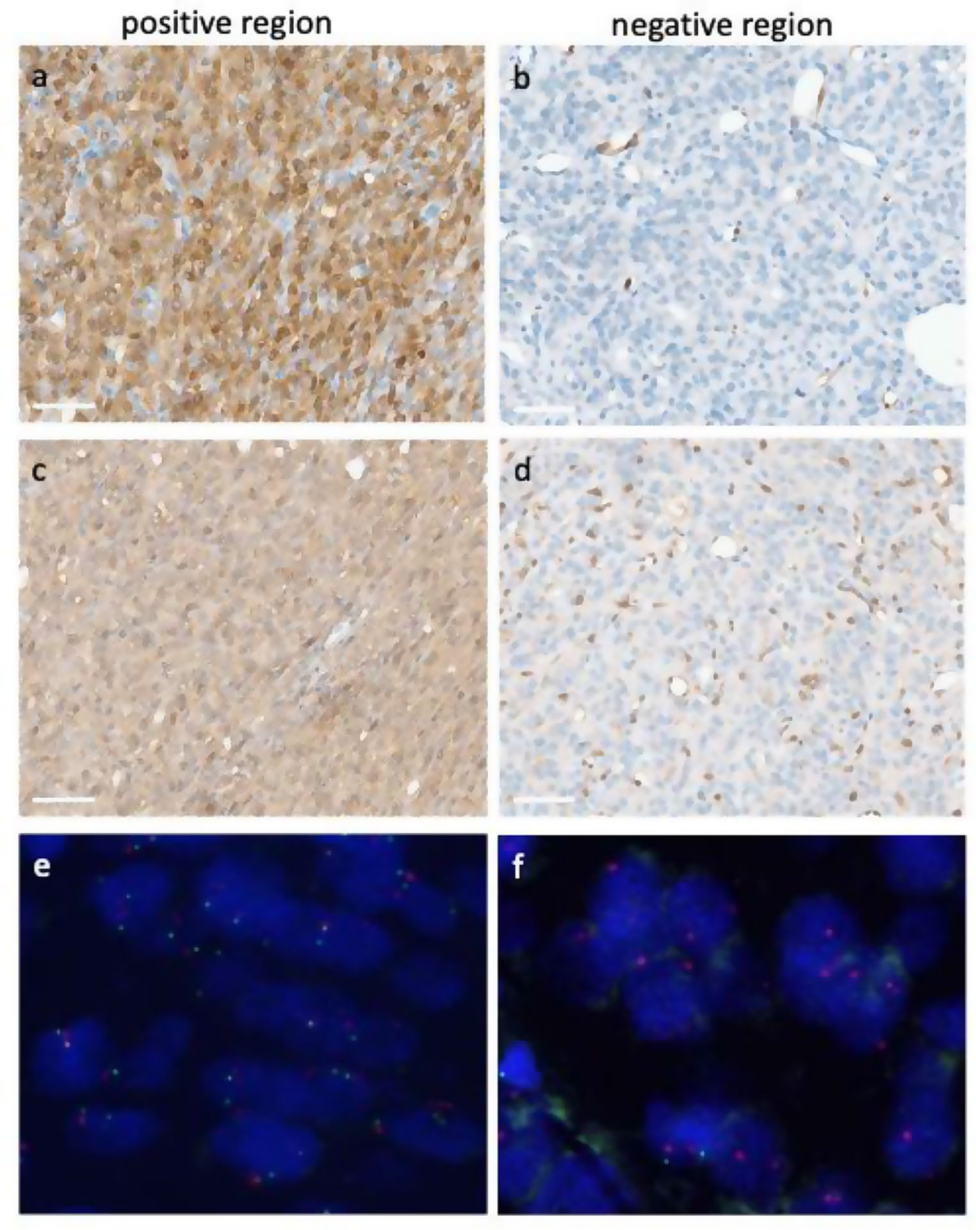
**a** p16 and **c** MTAP positive tumor region that was examined by FISH analysis and shown in **e** (Zytolight, SPEC CDKN2A/CEN 9 Dual Color Probe, Zytovision, Germany). **b** p16 and **d** MTAP negative tumor region. **f** FISH of this region lacking p16/MTAP expression. Green, *CDKN2A/MTAP*; red, centromere chromosome 9 (63x magnification). *scale bars* in a-d 60 μm

Additionally, FISH analysis (Zytolight) was performed on case #3 to determine whether the heterozygous *CDKN2A* deletion was clonal or indicative of a subclonal homozygous loss. Given the significant regional heterogeneity observed in the p16 and MTAP IHC, FISH was conducted on two distinct regions. The p16/MTAP-positive region exhibited both *CDKN2A/MTAP* and centromeric signals, while the region negative in IHC only showed the red centromeric signal (Fig. 2), thus indicating a subclonal homozygous loss in this region.

## Discussion

*CDKN2A* gene alterations are increasingly recognized for their role in tumor biology and prognosis. Since WHO grading of meningiomas has significant therapeutic implications, it is important to identify cases with an unfavorable molecular signature. As previously reported [21, 28], p16 IHC is a reliable screening tool for identifying cases that should undergo molecular analysis for *CDKN2A*. In the context of a higher-grade meningioma (e.g., elevated mitotic count, brain invasion, or other concerning features such as pattern loss, significantly increased proliferative activity, or necrosis) and p16/MTAP negativity, testing for a possible *CDKN2A* deletion is the next step. MTAP IHC is often used as a surrogate marker for *CDKN2A* deletions [14]. Several studies have compared the specificity and sensitivity of MTAP and p16 as markers for *CDKN2A* loss [16, 19]. In a large study involving 50 meningiomas, including eight WHO grade 3 cases [16], MTAP IHC demonstrated strong correlation with FISH results and outperformed p16 IHC. Notably, the FISH probe that was utilized in both studies (LSI *CDKN2A* FISH probe) encompassed both the *MTAP* and *CDKN2A/CDKN2B* regions, which may have led to the omission of cases with discordant *MTAP* and *CDKN2A* status. Regarding p16 IHC, it is important to note that non-proliferating cells or cells without cell cycle activation typically exhibit little to no p16 expression [21, 28]. Therefore, p16 positivity is not expected in low-proliferative lesions, such as CNS WHO grade 1 meningiomas. In our experience, if a meningioma exhibits concerning signs of atypia or anaplasia along with p16 immunonegativity, we would proceed with testing for *CDKN2A* alterations. Although MTAP staining offers the benefit of labeling normal cells as an internal (positive) control, our work shows that it is not always a dependable surrogate marker for *CDKN2A* status, particularly in anaplastic meningiomas with differing *CDKN2A* and *MTAP* statuses. In another series of 15 anaplastic meningioma, three tumors demonstrated a homozygous *CDKN2A* deletion, as shown by copy number variation (CNV) plots inferred from methylation analysis [22]. While two cases exhibited p16 negativity, a third case displayed only focal negativity of p16 expression. This observation may be attributed to spatial intratumoral clonal heterogeneity, with certain tumor regions harboring homozygous *CDKN2A* deletions, while others retain intact *CDKN2A* status [13, 19, 24]. In our cohort, we identified a case (case #3) exhibiting such subclonal homozygous loss, characterized by the absence of *CDKN2A/MTAP* deletion in one region and homozygous loss in another, as evidenced by a distinct regional p16/MTAP staining pattern. This finding aligns with previous reports of intratumoral genetic heterogeneity in meningioma [2, 13, 23]. Notably, since we performed MIP and methylation analyses on bulk DNA, a hemizygous loss was described. This underscores the importance of performing IHC for p16/MTAP prior to molecular analysis to identify cases exhibiting spatial heterogeneity. Molecular analyses in higher-grade meningiomas should then be performed on tumor regions showing no immunohistochemical staining for p16/MTAP.

We identified anaplastic meningiomas with homozygous *CDKN2A* deletions but retained *MTAP* gene and MTAP protein expression. These observations indicate that MTAP should not be relied upon as the sole surrogate immunohistochemical marker for *CDKN2A* deletions. Similarly, FISH probes that target a larger region containing both *CDKN2A* and *MTAP* should be avoided, as they may miss *CDKN2A* deletions by binding to *MTAP*, even if *CDKN2A* is homozygously deleted.

In addition to providing a methylation class estimate, DNA methylation analysis also allows the generation of a copy number profile. Whereas one can visually estimate the presence of homozygous or hemizygous *CDKN2A* deletions, this method does not allow adjustment for diploidy and is not quantitative; interobserver variability remains substantial. In contrast, a high-resolution, quantitative chromosomal copy-number array enables precise determination of deletion status. Additionally, breakpoints between *CDKN2A* and *MTAP* or within the *MTAP* gene can be identified. This method therefore provides a highly accurate estimation of the *CDKN2A* chromosomal status. Similarly, NGS allows precise estimation of the heterozygosity and could reliably detect homozygous deletions as well.

Alternative molecular mechanisms, such as mutations or hypermethylation, can result in the functional inactivation of the *CDKN2A* gene [4, 7, 15, 20]. In a large series of over 1500 meningiomas, Wang and colleagues found that heterozygous *CDKN2A/B* deletion had an impact on outcome as adverse as *CDKN2A* homozygous deletion. Interestingly, they also found a cohort of meningiomas with a high CDKN2A mRNA expression (CDKN2A^high^), which exhibited similarly aggressive outcomes to those with homozygous *CDKN2A* deletion and was enriched with transcriptomic pathways similar to those found in the *CDKN2A* homozygous loss group. Additionally, p16 expression closely correlated with CDKN2A mRNA levels [27]. These findings indicated that high CDKN2A mRNA expression could be a potential biomarker for clinical aggressiveness in meningiomas, especially in those lacking *CDKN2A* deletions.

Various molecular diagnostic methods are available to assess the *CDKN2A* copy number status. In this study, we demonstrate that in meningiomas, *CDKN2A* copy number status may differ from the *MTAP* status. This discrepancy can result in the missing of *CDKN2A* homozygous deletions and misgrading of meningiomas when MTAP IHC is used as a sole surrogate marker or when an inappropriate FISH probe is employed. The limited sample size in this cohort constrains the ability to draw generalizable conclusions. Therefore, additional studies involving larger cohorts are necessary to validate the findings of our research. Nevertheless, we propose that negative p16 immunostaining in higher-grade meningiomas suggests potential *CDKN2A* loss. Subsequent quantitative copy-number analysis can provide more precise diagnostics and enable accurate grading.

## Data Availability

The data that support the findings of this study are not openly available due to reasons of sensitivity and are available from the corresponding author upon reasonable request.
